# Identification of Novel FAM134B (JK1) Mutations in Oesophageal Squamous Cell Carcinoma

**DOI:** 10.1038/srep29173

**Published:** 2016-07-04

**Authors:** Md. Hakimul Haque, Vinod Gopalan, Kwok-wah Chan, Muhammad J. A. Shiddiky, Robert Anthony Smith, Alfred King-yin Lam

**Affiliations:** 1 Cancer Molecular Pathology in Menzies Health Institute Queensland, Griffith University, Gold Coast, Australia; 2 Department of Pathology, Li Ka Shing Faculty of Medicine, The University of Hong Kong, Queen Mary Hospital, Hong Kong; 3 School of Natural Sciences, Griffith University, Nathan Campus, Australia; 4 Genomics Research Centre, Institute of Health and Biomedical Innovation, Faculty of Health, Queensland University of Technology, Brisbane, Queensland, Australia

## Abstract

Mutation of *FAM134B* (*Family with Sequence Similarity 134, Member B*) leading to loss of function of its encoded Golgi protein and has been reported induce apoptosis in neurological disorders. *FAM134B* mutation is still unexplored in cancer. Herein, we studied the DNA copy number variation and novel mutation sites of *FAM134B* in a large cohort of freshly collected oesophageal squamous cell carcinoma (ESCC) tissue samples. In ESCC tissues, 37% (38/102) showed increased *FAM134B* DNA copies whereas 35% (36/102) showed loss of *FAM134B* copies relative to matched non-cancer tissues. Novel mutations were detected in exons 4, 5, 7, 9 as well as introns 2, 4-8 of *FAM134B via* HRM (High-Resolution Melt) and Sanger sequencing analysis. Overall, thirty-seven *FAM134B* mutations were noted in which most (31/37) mutations were homozygous. *FAM134B* mutations were detected in all the cases with metastatic ESCC in the lymph node tested and in 14% (8/57) of the primary ESCC. Genetic alteration of *FAM134B* is a frequent event in the progression of ESCCs. These findings imply that mutation might be the major driving source of *FAM134B* genetic modulation in ESCCs.

Oesophageal squamous cell carcinoma (ESCC) is the most common histological subtype of oesophageal carcinomas and it has complex molecular pathology compared to other carcinomas^1^,^2^,^3^. Identification of various genetic and epigenetic changes including mutations of key regulatory genes have a significant role in predicting the biological behaviour of ESCC as well as the prognosis of the patients with ESCC^4^,^5^,^6^,^7^,^8^,^9^,^10^,^11^,^12^,^13^.

Our previous studies using comparative genomic hybridization analysis revealed that genes in the region of chromosome 5p play a vital role in the pathogenesis of ESCC^14^,^15^. *FAM134B* (*Family with sequence similarity 134, member B*) also known as *JK1*is a novel gene placed at chromosome 5p15.1 downstream of δ-catenin^16^. The oncogenic properties of *FAM134B* (*JK1*) were first reported both in ESCC tissues and cell lines, while ESCC tissues also showed altered *FAM134B* expression^16^. Also, our recent studies have confirmed the growth related properties of *FAM134B* by exhibiting multiple tumour suppressor properties of *FAM134B* (*JK1*) in colorectal cancer tissues and cell lines^17^,^18^,^19^.

Recently, other researchers have reported that *FAM134B* encodes a *cis*-Golgi transmembrane protein, and its mutation can regulate cell apoptosis and long-term survival of nociceptive and autonomic ganglion neurons^20^,^21^,^22^,^23^,^24^,^25^. Homozygous loss of function mutations in *FAM134B* was reported for the first time in hereditary sensory and autonomic neuropathy type IIB (HSAN IIB) and in vascular dementia^20^,^24^. In addition, Khaminets *et al*. have most recently noted that *FAM134B* regulated endoplasmic reticulum turnover by selective autophagy^25^. To the best of our knowledge, at the time of writing, there is no data available on the mutational significance of *FAM134B* in human cancers. Also, the clinicopathological correlation of *FAM134B* mutation and clinicopathological parameters has never been reported in human cancer samples. Thus, the current study aims to detect mutations in different exon and intron regions of *FAM134B* (*JK1*) in ESCC tissue samples. In addition, the correlations of *FAM134B* (*JK1*) mutations with various clinicopathological parameters and natural copy number variations in ESCCs were analysed.

## Results

### Identification of *FAM134B* (*JK1*) DNA in ESCC tissues

**Figure 1:**
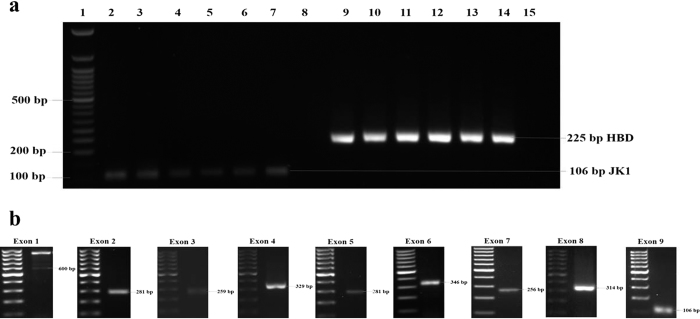
*FAM134B* (*JK1*) gene amplification in oesophageal squamous cell carcinoma. **(a)** Amplified PCR products of *FAM134B* (*JK1*) (122bp) and *HBD* (225bp) in 2% agarose gel. *FAM134B* (*JK1*) and *HBD* were present in all the samples (2–14) except for the water control (15). Fifty-base pair DNA ladder was used for comparison. **(b)** Representative amplified PCR products of exon 1, exon 2, exon 3, exon 4, exon 5, exon 6, exon 7, exon 8 and exon 9 of *FAM134B* (*JK1*) in 1.5% agarose gel. Hundred-base pair DNA ladder was used for comparison.

*FAM134B* (*JK1*) DNA was identified in all the studied samples. In these samples, 600-base pairs (bp), 281-bp, 259-bp, 329-bp, 282-bp, 346-bp, 256-bp, 314-bp and 106-bp fragments were amplified for exon 1, exon 2, exon 3, exon 4, exon 5, exon 6, exon 7, exon 8 and exon 9 of *FAM134B* (*JK1*) respectively. Also, a 225-bp fragment was amplified for the control gene, haemoglobin delta (HBD) (Fig. 1a,b).

### *FAM134B* (*JK1*) copy number variations

In ESCC (n = 102), 37% (n = 38) showed increased *FAM134B* (*JK1*) copies whereas 35% (n = 36) showed a loss of *FAM134B* (*JK1*) copies relative to matched non-cancer tissue. Meanwhile, the remaining 22% (n = 28) did not show any change in copies. In ESCCs with lymph node metastasis (n = 64), 66% (n = 42) showed high *FAM134B* (*JK1*) copies whereas 34% (n = 22) revealed deletion (p = 0.80). The DNA copy number of *FAM134B* (*JK1*) in cancer showed no statistical correlations with the grades and pathological stages of ESCC or with the gender and the age of the patient (p > 0.05) (Table 1).

### Mutation screening using high-resolution Melt (HRM) curve and Sanger sequencing

*FAM134B* (*JK1*) mutations showed no correlation with other clinical and pathological features of ESCC including gender, age of the patient as well as the sites, histological grades and pathological stages of carcinoma (p > 0.05) (Table 2). Also, mutations did not show any correlation with the DNA copy number of *FAM134B* (*JK1*) (Tables 3 and 4).

Overall, 37 *FAM134B* (*JK1*) mutations were found in ESCC (Tables 3 and 4). Of these, 36 *FAM134B* (*JK1*) mutations were noted in metastatic ESCC in lymph node and 16 mutations were detected in primary cancer. Of these 37 mutations, 6 were heterozygous mutations and 31 were homozygous mutations. On the other hand, only 14% (8/57) of the primary carcinoma harboured mutations of *FAM134B* (*JK1*). Mutations were noted in both the primary cancer and the lymph node metastasis with ESCC in 2 cases. In these 2 cases (cases 43 and 50), the mutations noted were identical. Also, of the 37 *FAM134B* (*JK1*) mutations, 11 mutations result in alteration of the amino acid sequences in *FAM134B* (*JK1*) protein while 26 mutations were synonymous.

**Figure 2:**
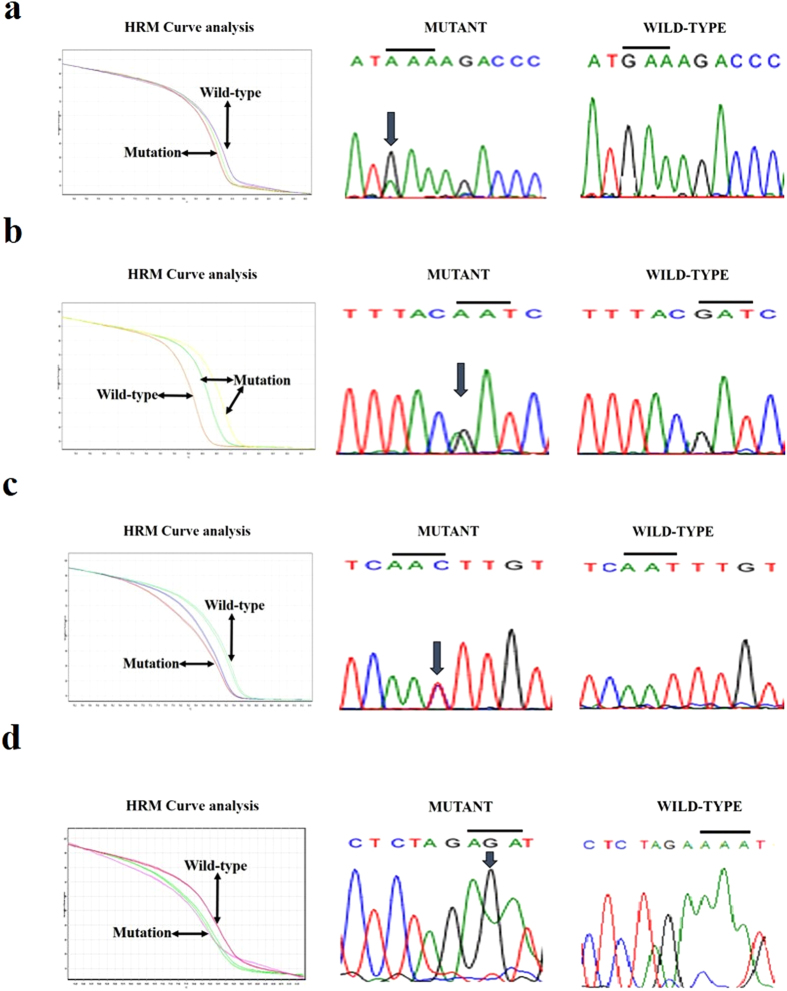
Validation of *FAM134B* (*JK1*) different exons sequence variant HRM curve analysis results by Sanger sequencing. **(a)** The presence of variant (GAA > AAA) in exon 4 of *FAM134B* (*JK1*) is demonstrated via normalized melting curves and sequencing (mutant versus wildtype). **(b)** HRM analysis shows the polymorphism (GAT > AAT) in exon 5 of *FAM134B* (*JK1*) as evident by normalized melting curves and sequencing (mutant versus wide type). **(c)** The presence of variant (AAT > AAC) in exon 7 of *FAM134B* (*JK1*) is confirmed via normalized melting curves and sequencing (mutant versus wide type). **(d)** The evidence of mutant (AAA > AGA) in exon 9 of FAM134B (JK1) is showed using normalized melting curves and sequencing (mutant versus wide type).

**Figure 3:**
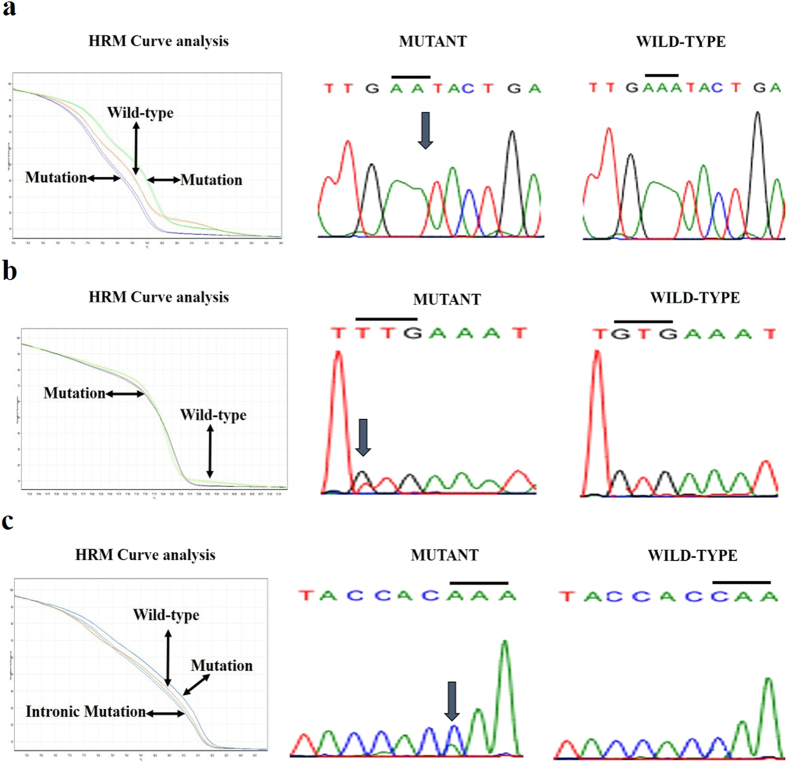
Confirmation of *FAM134B* (*JK1*) different introns sequence variant HRM curve analysis results via Sanger sequencing. **(a)** HRM curve analysis shows characteristic melting pattern of *FAM134B* (*JK1*) intron 2 amplicons using normalized melting curves and Sanger sequencing corroborates the mutation as being c. 14 + 144delA. **(b)** The evidence of mutant (GTG > TTG) in intron 6 of *FAM134B* (*JK1*) is shown using normalized melting curves and sequencing (mutant versus wild type). **(c)** HRM analysis shows the polymorphism (CAA > AAA) in intron 8 of *FAM134B* (*JK1*) as evident by normalized melting curves and sequencing (mutant versus wide type).

Among the 9 exons of *FAM134B* (*JK1*) examined, mutations were detected in exons 4, 5, 7 and 9 as well as introns 2, 4, 5, 6, 7 and 8 for *FAM134B* (*JK1*) in ESCC (Figs 2 and 3). In exon 9 of *FAM134B* (*JK1*), thirteen substitution and one frameshift (deletion) mutations were documented in ESCCs. The most common substitution variants in exon 9 are c. 1129T > C, c. 1107G > C, c. 1112T > C which alters the codons and subsequently amino acids - TCA > CCA (p. Ser348Pro), GAG > GAC (p. Glu340Asp) and GTT > GCT (p. Val342Ala) respectively. One frameshift (deletion) *FAM134B* (*JK1*) mutation, c. 1137delT, which alters the codon TTT > TTC resulting in no change in amino acid, but altered amino acids following the frameshift (p. Phe379fs) was noted in ESCC. For exon 4 of *FAM134B* (*JK1*), two mutations, c. 546-547CT > GG and c. 660G > A, were detected in ESCC tissues (Table 3). The mutation in c. 546-547CT > GG change the codons in amino acids - AGC > AGG (p. Ser153Arg) and TGG > GGG (p. Trp154Gly). Meanwhile the mutation in c. 660G > A change the codons in amino acids- GAA > AAA (p. Glu163Lys). Furthermore, four polymorphisms were in identified intron 4 *FAM134B* (*JK1*) in ESCC.

Mutations in other exons and introns of *FAM134B* (*JK1*) were less common in ESCC. There was no mutation noted in exon 8 of *FAM134B* (*JK1*) in ESCC. However, two types of variants, c961-33delA and c1087 + 97C > A, were detected in intron 8. Also, one silent mutation (c. 816C > T) and a mutation (c. 873 + 23T > C) were noted for exon 7 and intron 7 of *FAM134B* (*JK1*) in ESCC respectively. In addition, two synonymous variants (c. 319G > A and c. 352C > T) were noted in exon 5 of *FAM134B* in ESCC. Lastly, five polymorphisms were detected in intron 2 of *FAM134B* (with the most frequent variant being c. 408-27delA).

## Discussion

Chromosomal copy number changes can indicate activation of oncogenes and inactivation of tumour suppressor genes in human cancers^26^. In our previous studies, *FAM134B* (*JK1*) copy number alterations were found as a frequent event in colorectal adenoma and adenocarcinoma^19^. The current study is the first to investigate *FAM134B* (*JK1*) copy number changes in a large cohort of ESCC. Approximately one third of cancers studied revealed amplification and one third revealed deletion of *FAM134B* in ESCC. This altered DNA copy number changes of *FAM134B* indicate its varied modulation potential in different ESCC patients.

This current study is the first systematic study to investigate mutation sites in *FAM134B* (*JK1*) gene in ESCC. Until now, five homozygous mutations of *FAM134B* (*JK1*) were detected in neurological diseases. They are three nonsense mutations, one frame-shift mutation, and one is a splice-site mutation^20^,^21^,^22^,^23^. These mutations were located in exon 1 and intron 7 of the gene. In addition, there were 2 heterozygous mutations reported in exon 1. In ESCC, we have noted 37 distinct mutations of *FAM134B* (*JK1*). Thirty-one are homozygous mutations and 6 were heterozygous mutations. These mutations were found in exons 4, 5, 7, 9 and introns 2, 4, 5, 6, 7 and 8 of *FAM134B* (*JK1*). None of these mutation sites are identical to those noted reported in the neurological diseases. Thus, the mutations related to the pathogenesis of ESCC appear to be are unique. The roles of *FAM134B* (*JK1*) mutations in the pathogenesis of the cancer should therefore be different from their roles in neurological diseases. The mechanisms and presence of any downstream effects of the *FAM134B* (*JK1*) mutations need to be further investigated in ESCC to confirm their significance.

Of the 37 novel mutation sites in *FAM134B* (*JK1*) detected in ESCC, 11 mutations changed the amino acid of the resulting protein while 26 mutation sites do not alter the amino acid composition of the protein. The most common mutations are present in exon 9. Amongst the 11 mutations that changed the amino acid structure of JK1 are three relatively common variants in exon 9 (c. 1129T > C, c. 1107G > C, c. 1112T > C). Of these, c. 1129T > C, is predicted by *in silico* methods as being of minimal effect due to lack of conservation of this amino acid in other species while the other two have potentially higher functional significance due to their predicted ability to also cause splice site modification in the protein. In addition, in exon 9 of *FAM134B* (*JK1*), one frame-shift (deletion) mutation, c. 1137delT, is likely to have increased functional significance as it radically alters and can truncate the protein as well as potentially changing exon splicing of the RNA. The other important mutation that lead to an amino acid change was noted in exon 4 resulting in an exon change GAA > AAA (protein change-p.Glu163Lys). The novel mutations detected in this study that alters the amino acid structure of FAM134B (JK1) have potential to affect the function of *FAM134B* (*JK1*).

In the current study, *FAM134B* (*JK1*) mutations were noted predominately in metastatic ESCCs present in lymph nodes, implying that they have a role in the pathogenesis of lymph node metastases in ESCC. Mutation in the majority of metastatic lymph node tissues compared to primary tumours (Table 3) indicates that *FAM134B* (*JK1*) mutation may offer a survival advantage in different tumour microenvironments, or assist in colonisation of those particular microenvironments. Differing *FAM134B* (*JK1*) sequences imply that, the molecular interactions and characteristics would differ between primary and metastatic tumours. Furthermore, the classical model for metastatic processes suggests that the majority of the cancer cells in the primary tumour have low metastatic potential and only a few cells acquire enough somatic mutations to become metastatic^27^. Thus, the absence of *FAM134* (*JK1*) mutation in the bulk of primary ESCC tissues in this study might be due to the harvesting of cancer cells with fewer somatic mutations. Hence, it can be hypothesised that *FAM134B* (*JK1*) might act as a potential target for predicting lymph node metastasis in ESCC patients. Further research with a larger series of ESCC patients with matched metastatic tissues and potentially functional studies on particular mutations are needed to confirm the metastatic potential of *FAM134B* (*JK1*).

In conclusion, we identified for the first time the mutation of FAM134B (*JK1*) in ESCC tissue samples. The altered expression patterns and copy number variations of *FAM134B* in ESCCs might be modulated by these mutation changes in *FAM134B*. In this study, *FAM134B* mutations were frequently observed in metastatic lymph node tissues indicating its use as a potential predictor for metastasis in ESCCs. Multiple different genetic alterations incorporating mutations in coding sequences of *FAM134B* were observed and each might entail either alterations to expression or functional changes of this gene that could play a fundamental role in the progression of ESCCs.

## Methods

### Recruitment of tissue samples and clinicopathological data

Matched tumour samples and non-cancer tissue (near the surgical resection margin) from the same patient who underwent resection of ESCC were prospectively collected, snapped frozen in liquid nitrogen and stored in minus 80 °C by the author (AKL). Informed consent was obtained from all subjects. In addition, in each case, macroscopically enlarged lymph nodes suspicious for lymph node metastasis were also sampled, snap frozen and stored. After the collection, additional tissues blocks were taken, fixed in formalin and embedded in paraffin for pathological examination. Sections were then cut from these blocks and haematoxylin and eosin stained. They were studied by the author (AKL). The ESCCs were graded according to the World Health Organization (WHO) criteria^27^. The carcinomas were staged as per the TNM (tumour, lymph node and metastases) classification adopted in the American Joint Committee on Cancer^28^. Overall, tissues from 102 patients with ESCC were recruited. Eighteen patients had lymph nodes with metastatic ESCC sampled. For each patient, clinical and pathological parameters including gender and age of patients as well as the sites, grades, pathological stages of the ESCC were recorded.

### Genomic DNA extraction

Ethical approval was obtained for the use of these samples (GU Ref No: MED/19/08/HREC) by the Griffith University human research ethics committee. The methods were carried out in accordance with the approved guidelines.

The selected samples were sectioned using a cryostat (Leica CM 1850 UV, Wetzlar, Germany) and stained by haematoxylin and eosin. Light microscopic examination was performed by author, AKL, to confirm the presence of non-cancer and cancerous tissues for genomic DNA extraction. Five 10 μm slices was sectioned from the frozen tissue samples for DNA extraction. DNA was extracted and purified from frozen tissue samples using all prep DNA/RNA mini kit (Qiagen, Hilder, Germany), following manufacturer’s instructions. DNA quantification was accomplished via Nanodrop Spectrophotometer (BioLab, Ipswich, MA, USA) and purity was measured using 260/280 ratio. Concentration of DNA was noted in ng/μl and then stored at −20 °C until use.

### Primer design

Primers specific for determining *FAM134B* mutation and copy number changes were designed based on GenBank accession number for variant 1 NM_001034850 and for variant 2 NM_019000 as well as for a control gene, *HBD* (GenBank accession number NM_000519) using Primer3 version 0.4.0 (http://frodo.wi.mit.edu/primer3). All primers were analysed for specificity using Primer Blast (http://www.ncbi.nlm.nih.gov/tools/primer-blast) and Primer Premier program version 5 (Premier Biosoft, Palo Alto, CA, USA) to check for primer parameters like GC content, annealing temperature of primer and ΔG (Gibbs free energy change - energy required to break the secondary structure) and to forecast any possible mismatching, primer dimmer or hairpin formation.

Primers for *FAM134B* (*JK1*) gene were designed within the intronic regions on either side of the exon of interest to ensure coverage of the entire exon 1 to exon 9 to amplify and direct sequencing both isoforms of the gene for screening of mutations in ESCC. Primers were also designed for *FAM134B* (*JK1*) and control gene - *haemoglobin delta* (*HBD*) to identify DNA copy number variations in ESCC. The primer pairs were obtained from Sigma-Aldrich (St Louis, MO, USA). The list of chosen primer sets are summarized in Table 5.

### Real-time quantitative polymerase chain reaction (qPCR)

DNA copy number changes of *FAM134B* (*JK1*) were determined using a rotor Gene Q real-time PCR (RT-PCR) Detection system (Qiagen). RT-PCR was achieved in a total volume of 10 μl comprising 5 μl of 2XSensiMix SYBR No-ROX master mix (Bioline, London, UK), 1 μl of each 10 picomole/μl primer, 1 μl of cDNA/ genomic DNA at 20–50 ng/μl and 2 μl of Nuclease-free water. PCR cycling programs encompassed initial denaturation and activated the hot start DNA polymerase in one cycle of 7 minutes at 95 °C followed by 40 cycles of 10 seconds at 95 °C (denaturation), 30 seconds at 60 °C (annealing) and 20 seconds at 72 °C (extension). Melting curve analysis was carried out using 80 cycles of 30 seconds increasing from 55 °C. The melting curves of all final real-time PCR products were analysed for determination of genuine products and contamination by nonspecific products and primer dimers. All samples were also run on 2% agarose gel electrophoresis to ensure that the correct product was amplified in the reaction. To increase the reliability of the results, assays were accomplished in multiple replicates and a non-template control was included in all the experiment. The results of the quantitative real-time polymerase change reaction were analysed using methods published previously^29^.

### High-Resolution Melt (HRM) curve analysis

Fifty-seven matched cancer and non-cancer tissues (including 18 cases with lymph node metastases) were used for HRM analysis. Sections of the lymph nodes were confirmed to have lymph nodes metastases by histological examination as above. HRM curve analysis was accomplished by amplifying target sequences on the Rotor-Gene Q detection system (Qiagen) using the software Rotor-Gene ScreenClust HRM Software. The exon 1, exon 2, exon 3, exon 4, exon 5, exon 6, exon 7, exon 8, exon 9 of *FAM134B* (*JK1*) were PCR amplified in a total reaction volume of 10 μl comprising 5 μl of 2Xsensimix HRM master mix, 1 μl of 30 ng/μl genomic DNA, diethylpyrocarbonate (DEPC, RNase free) treated water 2 μl and 1 μl of 5 μmol/L JK-1 primer. The thermal cycling protocol started with one cycle of 98 °C for 4 minutes. Full activation of the SensiFAST DNA polymerase occurs within 30 seconds at 95 °C. This was followed by 40 cycles of 98 °C for 5 seconds. Then, the reaction mix was at annealed at 60 °C for 15 seconds in exon 2, exon 5, exon 6, exon 7, exon 8, exon 9; 64.5 °C for 15 seconds for exon 3 and exon 4 and at 72 °C for 15 seconds for exon 1. Each PCR run included a negative (no template) control. The melt curve data were generated by increasing the temperature from 65 °C to 85 °C for all assays, with a temperature increase rate of 0.05 °C/seconds and recording fluorescence at each step. All the reactions were done in duplicate to increase the reliability of the results. Each mutant allele had its own distinctive melting curve while compared to the wild-type allele. HRM results were interpreted as mutant when both replicates showed a variant compared to wild type. In cases of uncategorized samples, one replicate showed a variant and the other matched the wild type curve.

### Purification of PCR products and Sanger sequencing analysis

All the possible mutations detected by HRM analysis were further confirmed by Sanger sequencing. After HRM curve analysis, successful and specific PCR products showing one melting peak were purified according to the manufacturer’s protocols from the NucleoSpin Gel and PCR Clean-up kit (Macherey- Nagel, Bethlehem, PA, USA). The purified PCR products were subjected to sequence by corresponding forward and reverse primer using the Big Dye Terminator (BDT) chemistry Version 3.1 (Applied Biosystems, Foster City, CA, USA) under standardised cycling PCR conditions and analysed by the Australian Genome Research Facility (AGRF) using a 3730xl Capillary sequencer (Applied Biosystems). The composition of DNA sequencing reactions was followed according the samples preparation guide from AGRF. Sequence analysis was performed with Chromas 2.31 software.

### Statistical analysis

Correlations of *FAM134B* (*JK1*) copy number change and mutations with clinicopathological parameters were performed. Comparisons between groups were implemented using the chi-square test, likelihood ratio and Fisher’s exact test. All the data was entered into a computer data base and the statistical analysis was executed using the Statistical Package for Social Sciences for Windows (version 22.0, IBM SPSS Inc., New York, NY, USA). Significance level was taken at p < 0.05.

## Additional Information

**How to cite this article**: Haque, M. H. *et al*. Identification of Novel FAM134B (JK1) Mutations in Oesophageal Squamous Cell Carcinoma. *Sci. Rep.* **6**, 29173; doi: 10.1038/srep29173 (2016).

## Figures and Tables

**Table 1:** Clinicopathological features of ESCC and *JK1* (*FAM134B*) copy number variation.

Characteristics	Number of Patients	DNA copy			P value
		Amplification Deletion		No change	
Age					
<60 years	34(33.3%)	15(44.1%)	7(20.6%)	12(35.3%)	0.07
>60	68(66.7%)	23(33.8%)	29(42.6%)	16(23.5%)	
Gender					
Male	84(82.4%)	32(38.1%)	28(33.3%)	24(28.6%)	0.66
Female	18(17.6%)	6(33.3%)	8(44.4%)	4(22.2%)	
Histological Grade					
Well	22(21.6%)	10(45.5%)	6(27.3%)	6(27.3%)	0.61
Moderate	59(57.8%)	23(39.0%)	21(35.6%)	15(25.4%)	
Poor	21(20.6%)	5(23.8%)	9(42.9%)	7(33.3%)	
Site					
Upper	12(11.8%)	5(41.7%)	6(50.0%)	1(8.3%)	0.12
Middle	55(53.9%)	23(41.8%)	14(25.5%)	18(32.7%)	
Lower	35(34.3%)	10(28.6%)	16(45.7%)	9(25.7%)	
T Stage					
1	6(5.9%)	2(33.3%)	2(33.3%)	2(33.3%)	0.85
2	11(10.8%)	5(45.5%)	5(45.5%)	1(9.1%)	
3	64(62.7%)	23(35.9%)	22(34.4%)	19(29.7%)	
4	21(20.6%)	8(38.1%)	7(33.3%)	6(28.6%)	
Lymph node Metastasis					
Negative	38(37.3%)	11(28.9%)	14(36.8%)	13(34.2%)	0.33
Positive	64(62.7%)	27(42.2%)	22(34.4%)	15(23.4%)	
Distant metastasis					
Negative	97(95.1%)	35(36.1%)	35(36.1%)	27(27.8%)	0.55
Positive	5(4.9%)	3(60.0%)	1(20.0%)	1(20.0%)	
TNM Stage					
Stage I	6(5.9%)	4(66.7%)	0(0.0%)	2(33.3%)	0.22
Stage II	26(25.5%)	7(26.9%)	12(46.2%)	7(26.9%)	
Stage III	65(63.7%)	24(36.9%)	23(35.4%)	18(27.7%)	
Stage IV	5(4.9%)	3(60.0%)	1(20.0%)	1(20.0%)	

**Table 2:** Clinicopathological features and *JK1* (*FAM134B*) mutations in oesophageal squamous cell carcinoma.

Characteristics	Number of patients	Mutation cases	No mutation cases	p value
Age				
<60 year	20	11	9	0.17
>60 year	37	13	24	
Gender				
Male	50	19	31	0.12
Female	7	5	2	
Site				
Upper	5	2	3	0.44
Middle	40	15	25	
Lower	12	7	5	
Grade				
Well	20	7	13	0.58
Moderate	28	12	16	
Poor	9	5	4	
T Stage				
1	1	0	1	0.62
2	6	3	3	
3	38	17	21	
4	12	4	8	
Lymph node Metastasis				
Negative	20	7	13	0.58
Positive	37	17	20	
Distant metastasis				
Negative	54	22	32	0.57
Positive	3	2	1	
TNM Stage				
Stage I	1	1	0	0.55
Stage II	14	5	9	
Stage III	39	17	22	
Stage IV	3	2	1	

**Table 3:** Mutations detected in different exons of *FAM134B* (*JK1*) in ESCC.

Exons	Sample Code	Primary cancer with mutation	Lymph node metastasis with mutation	FAM134B DNA copy number change		DNA change	Type of mutation	Codon change	Protein change	Comments
				Primary cancer	Lymph node metastasis					
Exon 9	41	No	Yes	N	N	c. 1128T > A	Substitution	CTT > CTA	p.Leu347Leu	Potentially functional due to splice site modification
	30	No	Yes	D	D	c. 1107G > C c. 1129T > C	Substitution	GAG > GAC TCA > CCA	p.Glu340Asp p.Ser348Pro	pGlu340Asp - Likely functional significance pSer348Pro - Probably lesser effect/polymorphism due to lack of conservation in other species
	14	Yes	No	A	A	c. 1149A > G	Substitution	GAA > GAG	p.Glu354Glu	Likely functional significance (also affects splice sites)
	19	No	Yes	D	A	c. 1112T > C c. 1129T > C	Substitution	GTT > GCT TCA > CCA	p.Val342Ala p.Ser348Pro	pVal342Ala - Likely functional significance (also affects splice sites) pSer348Pro - Probably lesser effect/polymorphism due to lack of conservation in other species
	3	Yes	No	D	D	c. 1112T > C c. 1149A > G c. 1152T > G c. 1153G > C	Substitution	GTT > GCT GAA > GAG AAT > AAG GGC > CGC	p.Val342Ala p.Glu354Glu p.Asn355Lys p.Gly356Arg	pVal342Ala - Likely functional significance (also affects splice sites) pGlu354Glu - Likely functional significance (also affects splice sites) pAsn355Lys - Likely functional significance pGly356Arg - Likely functional significance (also affects splice sites)
	35	Yes	No	A	–	c. 1092T > G c. 1093G > C c. 1097GA > AC c. 1107G > T c. 1129T > C	Substitution	CTT > CTG GAC > CAC CGA > CAC GAG > GAC TCA > CCA	p.Leu335Leu p.Asp336His p.Arg337His p.Glu340Asp p.Ser348Pro	pLeu335Leu - Likely functional significance (also affects splice sites) pAsp336His- Likely functional significance (also affects splice sites) pArg337His - Likely functional significance (also affects splice sites) pGlu340Asp - Likely functional significance pSer348Pro - Probably lesser effect/polymorphism due to lack of conservation in other species
	43	Yes	Yes	A	N	c. 1112T > C c. 1129T > C c. 1092T > G c. 1093G > C	Substitution	GTT > GCT TCA > CCA CTT > CTG GAC > CAC	p.Val342Ala p.Ser348Pro p.Leu335Leu p.Asp336His	pVal342Ala - Likely functional significance (also affects splice sites) pSer348Pro - Probably lesser effect/polymorphism due to lack of conservation in other species pLeu335Leu - Likely functional significance (also affects splice sites) pAsp336His- Likely functional significance (also affects splice sites)
	47	Yes	No	D	–	c. 1107G > C c. 1129T > C	Substitution	GAG > GAC TCA > CCA	p.Glu340Asp p.Ser348Pro	pGlu340Asp - Likely functional significance pSer348Pro - Probably lesser effect/polymorphism due to lack of conservation in other species
	50	Yes	Yes	D	A	c. 1107G > T c. 1129T > C c. 1164A > G	Substitution	GAG > GAC TCA > CCA ACA > ACG	p.Glu340Asp p.Ser348Pro p.Thr360Thr	pGlu340Asp - Likely functional significance pSer348Pro - Probably lesser effect/polymorphism due to lack of conservation in other species pThr360Thr -Likely functional significance (also affects splice sites)
	51	Yes	No	N	–	c. 1112T > C c. 1129T > C	Substitution	GTT > GCT TCA > CCA	p.Val342Ala p.Ser348Pro	pVal342Ala - Likely functional significance (also affects splice sites) pSer348Pro - Probably lesser effect/polymorphism due to lack of conservation in other species
	34	Yes	No	A	–	c. 1137delT	Deletion	TTT > TTC	p.Phe350Phe	pPhe350Phe- Likely functional significance (frameshift mutation, truncates protein, may change splicing)
Exon 7	39	No	Yes	A	N	c. 816C > T or c. 903C > T	Substitution	GAT > GAC	p.Asp272Asp	pAsp272Asp-Likely functional significance (also affects splice sites)
	19	No	Yes	D	A	c. 816C > T or c. 903C > T	Substitution	GAT > GAC	p.Asp272Asp	pAsp272Asp -Likely functional significance (also affects splice sites)
Exon 5	14	Yes	No	A	A	c. 711G > Ac. 744C > T	Substitution Substitution	ACG > ACA CTC > CTT	p.Thr208Thr p.Leu219Leu	Has no functional significance Has no functional significance
	6	No	Yes	A	A	c. 711G > A	Substitution	ACG > ACA	p.Thr208Thr	Has no functional significance
	12	No	Yes	N	N	c. 711G > A	Substitution	ACG > ACA	p.Thr208Thr	Has no functional significance
	41	No	Yes	N	N	c. 711G > A	Substitution	ACG > ACA	p.Thr208Thr	Has no functional significance
	20	No	Yes	N	N	c. 711G > A	Substitution	ACG > ACA	p.Thr208Thr	Has no functional significance
	11	No	Yes	A	N	c. 711G > A	Substitution	ACG > ACA	p.Thr208Thr	Has no functional significance
	3	Yes	No	D	D	c. 711G > A	Substitution	ACG > ACA	p.Thr208Thr	Has no functional significance
	15	No	Yes	D	N	c. 711G > A	Substitution	ACG > ACA	p.Thr208Thr	Has no functional significance
Exon 4	6	No	Yes	A	A	c. 546–547CT > GG c. 660G > A	Substitution Substitution	AGC > AGG and TGG > GGG GAA > AAA	p.Ser153Arg p.Trp154Gly p.Glu163Lys	pSer153Arg and pTrp154Gly-Likely functional significance. (also affects splice sites) pGlu163Lys-Likely functional significance (also affects splice sites).
	38	No	Yes	N	D	c. 660G > A	Substitution	GAA > AAA	p.Glu163Lys	pGlu163Lys-Likely functional significance (also affects splice sites).
	30	No	Yes	D	D	c. 660G > A	Substitution	GAA > AAA	p.Glu163Lys	pGlu163Lys-Likely functional significance (also affects splice sites).
	43	No	Yes	A	N	c. 660G > A	Substitution	GAA > AAA	p.Glu163Lys	pGlu163Lys-Likely functional significance (also affects splice sites).
	59	No	Yes	N	D	c. 660G > A	Substitution	GAA > AAA	p.Glu163Lys	pGlu163Lys-Likely functional significance (also affects splice sites).
	19	No	Yes	D	A	c. 660G > A	Substitution	GAA > AAA	p.Glu163Lys	pGlu163Lys-Likely functional significance (also affects splice sites).

**Table 4:** Mutations detected in different introns of *FAM134B* (*JK1*) in ESCC.

Introns	Sample Code	Primary cancer	Lymph node metastasis	***FAM134B*** **DNA copy number change**		DNA change	Type of mutation
				Primary cancer	Lymph node metastasis		
Intron 2	39	No	Yes	A	N	c. 514 + 144delA c. 514 + 37 − 38delTT	Deletion Deletion
	41	No	Yes	N	N	c. 408 − 27delA c. 514 + 37 − 38delTT	Deletion Deletion
	20	No	Yes	N	N	c. 408 − 27delA c. 514 + 34T > A c. 514 + 37 − 38delTT	Deletion Substitution Deletion
	73	No	Yes	A	A	c. 408 − 27delA	Deletion
Intron 4	16	No	Yes	N	D	c. 408 − 27delA c. 514 + 37 − 38delTT	Deletion Deletion
	39	No	Yes	A	N	c. 546 − 64delA c. 672 + 46 − 47delTG	Deletion Deletion
	6	No	Yes	A	A	c. 546 − 64delA c. 546 − 56delA c. 672 + 46 − 47delTG	Deletion Deletion Deletion
	38	No	Yes	N	D	c. 546 − 56delA c. 672 + 26delT	Deletion Deletion
	30	No	Yes	D	D	c. 546 − 64delA c. 546 − 56delA	Deletion Deletion
Intron 5	43	No	Yes	A	N	c. 546 − 56delA c. 672 + 26delT	Deletion Deletion
	59	No	Yes	N	D	c. 546 − 56delA c. 672 + 26delT c. 672 + 46 − 47delTG	Deletion Deletion Deletion
	19	No	Yes	D	A	c. 546 − 56delA	Deletion
	41	No	Yes	N	N	c. 673 − 54delC	Deletion
	11	No	Yes	A	N	c. 673 − 54C > A c. 757 + 56G > A	Substitution Substitution
	3	Yes	No	D	D	c. 673 − 65delA	Deletion
	15	No	Yes	D	N	c. 757 + 56G > A c. 673 − 52 − 53GA > AG	Substitution Substitution
Intron 6	38	No	Yes	N	D	c. 758 − 61 − 62TG > CTc. 758 − 71G > T	Substitution Substitution
	30	No	Yes	D	D	c. 758 − 71G > T	Substitution
Intron 7	19	No	Yes	D	A	c. 873 + 23T > C or c. 960 + 23T > C	Substitution
Intron 8	14	Yes	No	A	A	c. 1087 + 97C > A	Substitution
	6	No	Yes	A	A	c. 1087 + 97C > A	Substitution
	30	No	Yes	D	D	c. 961 − 33delA c. 1087 + 97C > A	Deletion Substitution
	41	No	Yes	N	N	c. 961 − 33delA c. 1087 + 97C > A	Deletion Substitution
	31	No	Yes	A	A	c. 961 − 33delA c. 1087 + 97C > A	Deletion Substitution

**Table 5:** List of Exon primers designed for qRT-PCR and sequencing of *FAM134B*.

Target Genes	Primer sequence (Forward and Reverse)	Amplicon size
*FAM134B* Exon 1	5′-CGGCACCCACACCCAGGCGCGCCC-3′	600 bp
	5′-GCACTGGGTCCCCGGGGCCCCG-3′	
*FAM134B* Exon 2	5′-GTTTCTGTGGCAGGAAGTAAACCC-3′	281 bp
	5′-GGACTAATTGGCTAATATGCCTAC-3′	
*FAM134B* Exon 3	5′-GTGTTAGAGATCTGAGCATTCCAC-3′	259 bp
	5′-CTTGGATTTAGATTCCTGTCAC-3′	
*FAM134B* Exon 4	5′-CCAGAGGGTGTGGCCAACAGTAG-3′	329 bp
	5′-TGGAGAAATCTGACAAGCTG-3′	
*FAM134B* Exon 5	5′-GTCACTTATACCGGTCACTATAG-3′	282 bp
	5′-CTCATCCCCCCTCTTCAAAC-3′	
*FAM134B* Exon 6	5′-GTGAAATACTGAAATGTACGTAGC-3′	346 bp
	5′-CTTTGAGGGAGATTAGCTTC-3′	
*FAM134B* Exon 7	5′-GAATATGAGAAATGTGGGGTAAG-3′	256 bp
	5′-GGAGTTTATTAGGAAGATCATTCAGC-3′	
*FAM134B* Exon 8	5′-CTGTCATTTTGGGGGTTCATATGG-3′	314 bp
	5′-TGGTGGTAACATGTTATTTACCC-3′	106 bp
*FAM134B* Exon 9	5′-TGACCGACCCAGTGAGGA-3′	
	5′-GGGCAAACCAAGGCTTAA-3′	
*Haemoglobin delta*	5′-TGGATGAAGTTGGTGGTGAG-3′	225 bp
(HBD)	5′-CAGCATCAGGAGTGGACAGA-3′	
